# Determination of Aniline and Its Derivatives in Environmental Water by Capillary Electrophoresis with On-Line Concentration

**DOI:** 10.3390/ijms13066863

**Published:** 2012-06-05

**Authors:** Shuhui Liu, Wenjun Wang, Jie Chen, Jianzhi Sun

**Affiliations:** 1 College of Sciences, Northwest A & F University, Yangling, Shaanxi 712100, China; E-Mails: wang3108@126.com (W.W.); baifk1988@nwsuaf.edu.cn (J.C.); sjz06101959@yeah.net (J.S.); 2 State Key Laboratory of Crop Stress Biology in Arid Areas, Yangling 712100, China

**Keywords:** capillary electrophoresis, water analysis, environmental analysis, aromatic amines, on-line concentration

## Abstract

This paper describes a simple, sensitive and environmentally benign method for the direct determination of aniline and its derivatives in environmental water samples by capillary zone electrophoresis (CZE) with field-enhanced sample injection. The parameters that influenced the enhancement and separation efficiencies were investigated. Surprisingly, under the optimized conditions, two linear ranges for the calibration plot, 1–50 ng/mL and 50–1000 ng/mL (*R* > 0.998), were obtained. The detection limit was in the range of 0.29–0.43 ng/mL. To eliminate the effect of the real sample matrix on the stacking efficiency, the standard addition method was applied to the analysis of water samples from local rivers.

## 1. Introduction

Aniline and its derivatives are important raw materials in the pesticide, plastic and pharmaceutical industries. Their considerable consumption in industrial processes has led to a release of chemical by-products into aquatic environments. These compounds are well known due to their high toxicity and suspected carcinogenicity [1–4].

The most common techniques for the determination of aniline and its derivatives in environmental samples have been and remain GC [5,6] and HPLC [7,8]. Due to the low concentration of aniline and its derivatives in real samples, most methods rely on some types of pretreatment of the samples before analysis. Liquid-liquid extraction and solid-phase extraction have been frequently adopted, which are time-consuming and harmful to the environment due to the large volume of organic solvents used. There are few methods that can be employed to directly analyze aniline and its derivatives in environmental water. Varney [9] and Sun [10] achieved a limit of detection (LOD) of 1.5–15 nM by HPLC and 10 nM by CE, respectively, using electrochemical detection, which is not a common chromatographic technique. Although Chen obtained a LOD in the range of 0.1–10 nM using CE with on-line stacking [11], this concentration method was effective only for those aniline and its derivatives that have a high degree of association with micelle structures.

After development over a period of decades, CE has become a mature technique for analytical separation [12–14]. CE possesses several advantages over other separation techniques, including high separation efficiency, minimal sample requirements, and almost no need for organic solvents, which makes CE an environmentally friendly separation technique. Due to its poor concentration sensitivity with photometric detectors, CE has yet to be widely accepted within environmental field as a routine analytical method. However, for compounds such as aniline and its derivatives, CE is suitable as a chromatographic separation technique because the detection sensitivity can be easily improved by field-enhanced sample injection [15–18]. Furthermore, this on-line concentration method only works for cationic analytes, not for non-ionic or anionic analytes, resulting in high sensitivity and intrinsic selectivity for the final, overall analysis.

## 2. Results and Discussion

### 2.1. Development of Separation Parameters

**Figure 1:**
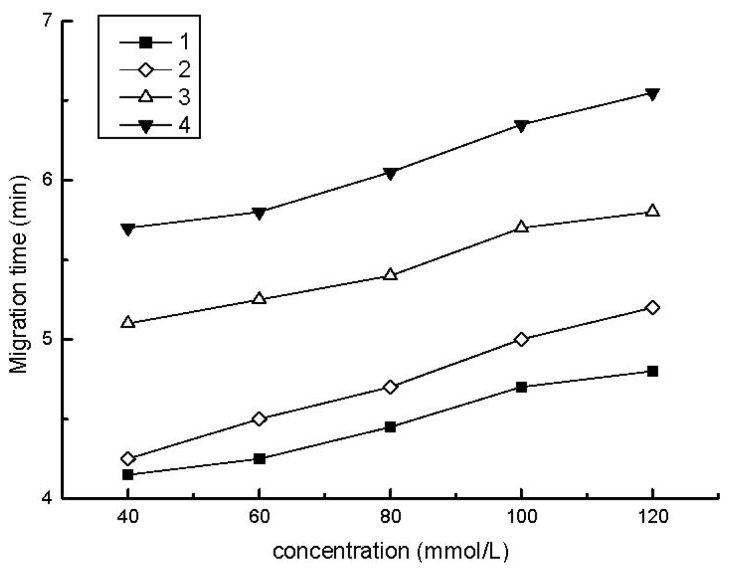
Effect of buffer concentration on the migration times of the analytes. ■: aniline; ⋄: *o*-tolidine; △: *o*-toluidine; ▼: *m*-aminophenol. Background electrolyte (BGE): varying amounts of NaH_2_PO_4_ and 10 mM triethanolamine, pH 2.6; sample concentration: 50 ng/mL each in 2 mM phosphoric acid and 60% acetonitrile; electroinjection: 6 s at 10 kV after injection of a water plug for 5 s at 0.5 psi.

The first experimental variable examined was the buffer concentration. In all cases, phosphoric acid/sodium dihydrogen phosphate was used as the electrolyte buffer because successful separation results were obtained. Figure 1 illustrates the effect of the buffer concentration on the migration times of the analytes. As the buffer concentration was increased, the overall migration times slightly increased due to the associated decrease in the electroosmotic flow through the column.

The peak height of each analyte increased as the buffer concentration increased due to the enhancement in the electric field amplification at the higher buffer concentrations. When the buffer concentration was greater than 120 mM, zone broadening occurred due to significant Joule heating effects. As a compromise between analyte resolution and concentration factors, a 120 mM phosphate buffer was selected for all further experiments. In addition, 10 mM triethanolamine was added to the buffer solution to decrease absorption of the analytes onto the capillary wall, and 2 mM phosphoric acid was employed in the sample solution to protonate the analytes and to maintain stable electroinjection conditions.

**Figure 2:**
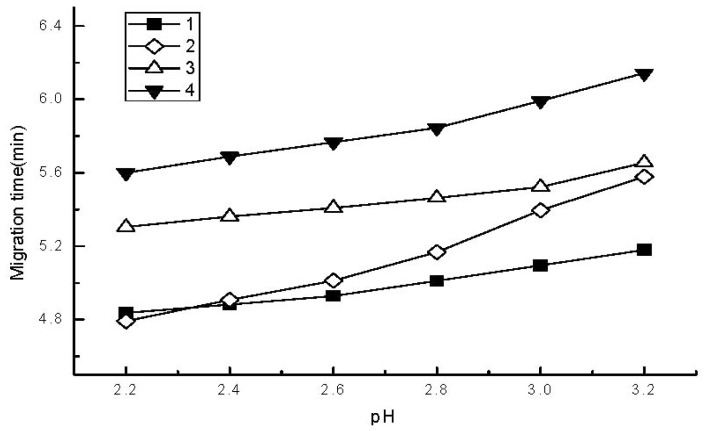
Effect of buffer pH on the migration times of the analytes. BGE, 80 mM of NaH_2_PO_4_ and 10 mM triethanolamine; for other experimental conditions, see Figure 1.

The effect of the buffer solution pH on the migration time of the analytes is shown in Figure 2. The migration order of the four analytes depended mainly on their p*K*_b_ (*o*-tolidine, p*K*_b1_ = 8.21, p*K*_b2_ = 9.54; aniline, p*K*_b_ = 9.39; o-toluidine, p*K*_b_ = 9.56; m-aminophenol, p*K*_b_ = 9.70). At low pH, the diamine compound (1) had the highest mobility due to the lowest p*K*_b_, and at higher pH, the migration sequence between aniline and *o*-tolidine was reversed. The increase in pH caused an increase in the migration time without a significant effect on analyte peak heights. A pH value of 2.8 was selected for routine work.

### 2.2. Optimization of Field-Enhanced Sample Injection

In field-enhanced sample injection, an electrokinetic injection was usually used to obtain higher detection sensitivity compared to hydrodynamic injection. A short plug of water, before electrokinetic injection of the sample, provided an appropriate enhancement of the electric field at the injection point, which produced an empty region for the concentration of ions deeper into the column and away from the inlet end. As discussed in our previous study [19], the water plug was necessary to maintain good reproducibility of the peak heights or areas. In the present study, the injection time of the water plug was 5 s.

#### 2.2.1. Effect of Sample Injection Time and Voltage

**Figure 3:**
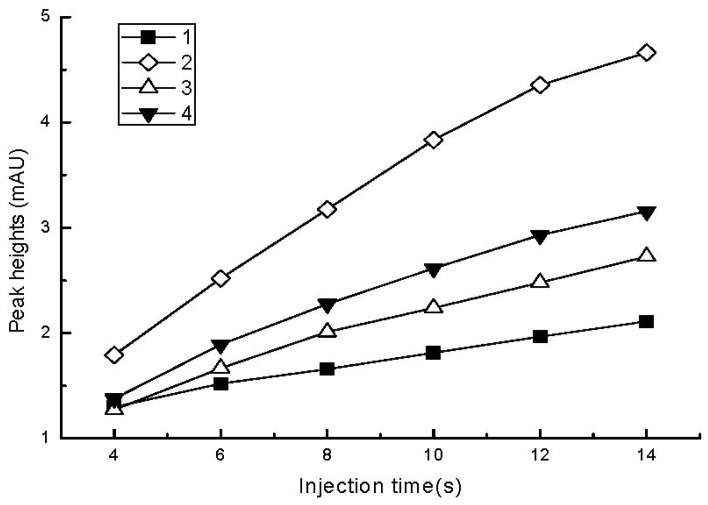
Effect of the injection time on the peak heights of the analytes. Injection voltage: 8 kV; other conditions are the same as in Figure 1.

As shown in Figure 3, the peak heights of the analytes increased rapidly as the injection time increased from 4 to 12 s. With a further increase in injection time, the peak heights changed slightly and the peaks were broadened. Therefore, an injection time of 12 s was optimal for the standard solutions.

**Figure 4:**
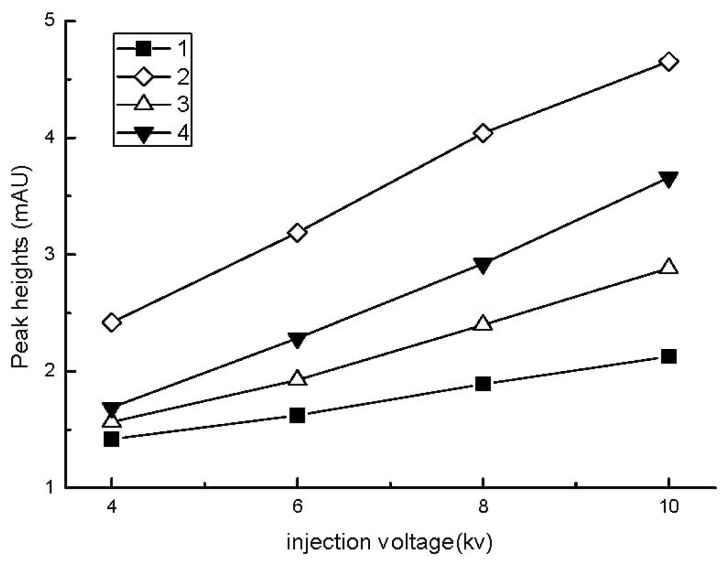
Effect of injection voltage on the peak heights of the analytes. Electrokinetic injection time: 12 s; other conditions are the same as in Figure 1.

The effect of the injection voltage on the peak heights of the analytes was similar to the injection time (Figure 4). Analyte ions permeated the boundary between the water plug and the run buffer into the buffer if longer injection times or higher voltages were applied, and the sample zones became dispersed, which resulted in an incomplete stacking process. The data presented in Figure 4 illustrate that the effective injection voltage was 8 kV in this study.

#### 2.2.2. Effect of Different Organic Solvents on Sample Stacking

**Figure 5:**
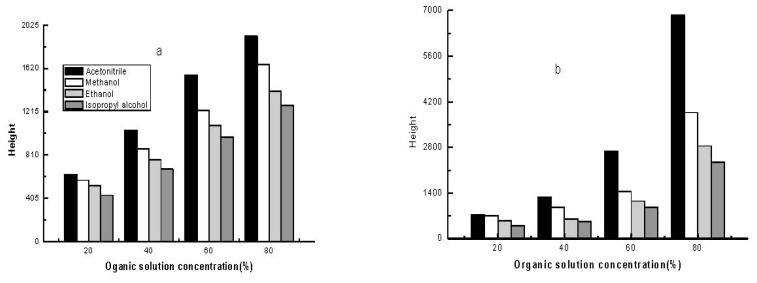

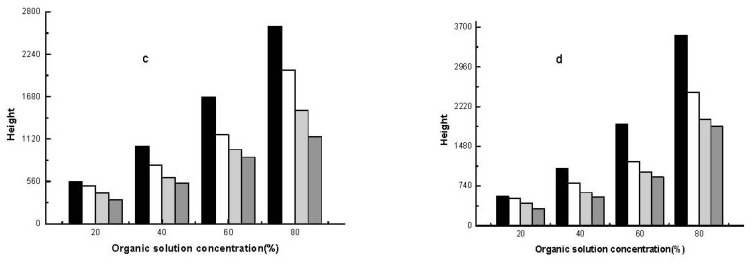
Effect of different organic solvents on stacking efficiency of the analytes by electroinjection 6 s at 8 kv: (**a**) aniline; (**b**) *o*-tolidine; (**c**) *o*-toluidine; (**d**) *m*-amonophenol. For other experimental conditions see Figure 1.

The effect of the volume ratio of different organic solvents on sample stacking is shown in Figure 5. As the percentage of solvent was increased, the peak heights of the analytes increased. At the same volume ratio for each organic solvent tested, the use of acetonitrile resulted in the highest stacking efficiency. A volume ratio of 60% acetonitrile was adopted because the shapes of the analyte peaks in the real samples deteriorated at 80% acetonitrile, which was probably due to a low conductivity of the sample [20].

Since the real sample matrix had a complicated composition, which influenced the on-line stacking efficiency, the standard addition method was used for quantitative analysis [21]. When the optimized stacking parameters were applied to the standard solutions spiked with local river water, a distortion in the peak shape occurred. After the stacking, parameters for real samples were further investigated, and the optimal injection time and voltage were determined to be 6 s and 8 kV, respectively.

### 2.3. Reproducibility, Linearity and Detection Limits

Linearity for all of the tested amine compounds was verified by studying different concentrations of each amine over a range of 1–1000 ng/mL. Interestingly, two linear trends appeared within the investigated concentration range; one trend occurred at low concentrations, over a range of 5–50 ng/mL (for aniline, 1–50 ng/mL), and another trend occurred at higher concentrations, over a range of 50–1000 ng/mL (for *o*-tolidine, 5–800 ng/mL) (Table 1). Regression coefficients and characteristics of the calibration plots were calculated. As illustrated, good linearity for all of the amines was achieved over both of these concentration ranges. The limit of detection ranged from 0.29 to 0.44 ng/mL with a signal-to-noise ratio of 3. The reproducibility of this method was validated in terms of the migration times and the peak heights of the analytes. For a given sample, RSD values of the migration times and peak heights for five replicate injections were below 0.5% and 4.5%, respectively.

### 2.4. Real Sample Analysis and Recovery

**Figure 6:**
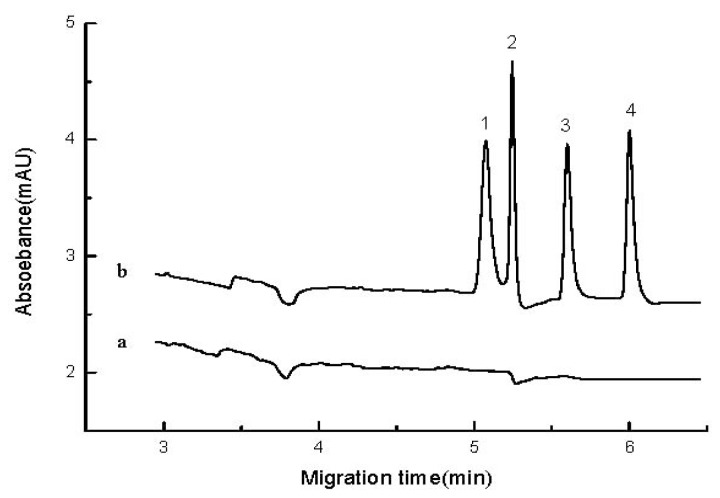
Electropherograms of the gaogan river sample and the 50 ng/mL internal standard analytes of gaogan river sample (**a**) 10% gaogan river sample (0.002 mol/L, phosphoric acid, 60% acetonitrile); (**b**) 50 ng/mL internal standard analytes of sample (other condition are the same as a).

The developed method was applied to the determination of aniline and its derivatives in samples collected from three local rivers (Yangling, China). To eliminate the effect of the real sample matrix on stacking efficiency, a standard addition method was used for quantitative analysis (Figure 6). A volume ratio of 10% river water was added into volumetric flasks that contained standard solutions with the specified concentration. The four tested compounds were not found in the real samples. Recoveries were calculated from the Gaogan Qu River sample spiked with aromatic amine standards, and the results were satisfactory (Table 2).

## 3. Experimental Section

### 3.1. Chemicals

Analytical grade sodium hydroxide, sodium dihydrogen phosphate, triethanolamine, and phosphoric acid were obtained from Tianli Chemical Reagent Co. Ltd. (Tianjing, China). HPLC grade acetonitrile, acetone, ethanol, and methanol were purchased from Luomiou Chemical Reagent Co. Ltd. (Tianjing, China). Aniline, *o*-tolidine, *o*-toluidine, and *m*-aminophenol were purchased from Sigma-Aldrich (St. Louis, MO, USA).

Stock solutions of analyte standards (5 mg/mL) were prepared in methanol and stored in a refrigerator at 4 °C. Fresh standard solutions were prepared daily by suitable dilutions of the stock solutions to prevent possible degradation of the analytes.

### 3.2. Apparatus

All experiments were performed on a Beckman P/ACE™ MDQ Capillary Electrophoresis System equipped with a photodiode array detector (PDA). The system was controlled by System Gold software, version 8.1 (Beckman Coulter Inc.: Fullerton, CA, USA, 2006). A fused silica capillary of 57 cm × 50 μm i.d. (50 cm to the detector), manufactured by Ruipu Chromatogram Equipment Company (Yongnian, China), was maintained at 25 °C in a cartridge with a 100 × 800 μm detection window. The PDA acquisition range was set between 190 and 300 nm (bandwidth of 6 nm) at a spectral acquisition rate of 4 Hz. Electropherograms were recorded at 214 nm. The pH of all solutions was measured using pH 213 equipment (Hanna Instruments, Baranzate, Italy). Deionised (18.2 MΩ) water was obtained using a Millipore Direct-Q 3 system (Millipore Corporation, Bedford, MA, USA).

### 3.3. Experimental Procedure

To activate a new capillary, it was rinsed with 0.1 M NaOH for 30 min, deionized water for 30 min, and a background electrolyte (BGE) solution for 30 min. On a daily basis, prior to the first sample injection, the capillary was flushed with 0.1 M NaOH for 3 min, deionized water for 2 min, and BGE for 5 min. Samples were introduced from the anodic end of the capillary by electroinjection using the specified voltage and time. All BGEs were degassed by sonication and filtered through 0.22-μm mixed cellulose ester filters (Shanghai Minimo Separation Technology Co. Ltd., Shanghai, China).

### 3.4. Preparation of Real Samples

Water samples were collected from three local rivers (Gaogan Qu River, Weihui Qu River and Suixi Gou River; Yangling, China), and the samples were analyzed after filtering through 0.45-μm cellulose ester filters.

## 4. Conclusions

The proposed method for determining amine compounds in environmental water samples by capillary zone electrophoresis (CZE) with on-line concentration is simple and efficient. The selectivity of this technique limits interference from differently charged compounds in complex matrices, which simplifies a tedious procedure for sample pretreatment. In addition, this method provides the required sensitivity for the inherent toxicity and the possible dangers of these compounds in environmental waters. Finally, it consumes low volumes of organic solvents (a few milliliters) and is relatively environmentally benign. This approach should find widespread applicability in this field in terms of environmental protection issues.

## Figures and Tables

**Table 1:** Parameters of calibration graphs and limits of detection for aromatic amines.

Compound	Regression Equation	Correlation Coefficient	Linear Range (ng/mL)	Detection Limit (ng/mL)
Aniline	y = 40.8x + 50.061	0.9983	1~50	0.29
	y = 5.1371x + 1149.9	0.9995	50~1000	

*o*-Tolidine; *o*-Toluidine	y = 30.588x + 324.09	0.9996	5~800	0.39
	y = 27.016x + 16.356	0.9999	5~50	0.44
	y = 9.9569x + 907.81	0.9993	50~800	

3-Aminophenol	y = 27.895x + 49.048	0.9997	5~50	0.43
	y = 12.142x + 769.55	0.9994	50~1000	

**Table 2:** Recovery of aromatic amines from Gaogan water samples.

Compound	Analytes Spiked (ng/mL)	Measured Amount (ng/mL)	RSD (%)	Recovery (%)
Aniline	20	20.8	4.93	104
	300	278.2	3.60	93

*o*-Tolidine	20	19.2	1.69	96
	300	290.8	5.85	97

*o*-Toluidine	20	19.4	4.37	97
	300	279.8	4.11	93

*m*-Aminophenol	20	18.9	2.79	95
	300	294.5	5.17	98
